# Positive Pacing Strategies Are Utilized by Elite Male and Female Para-cyclists in Short Time Trials in the Velodrome

**DOI:** 10.3389/fphys.2015.00425

**Published:** 2016-01-18

**Authors:** Rachel L. Wright

**Affiliations:** School of Sport, Exercise and Rehabilitation Sciences, College of Life and Environmental Sciences, University of BirminghamBirmingham, UK

**Keywords:** para-cycling, pacing, time trial, performance, Paralympic, velodrome, cycling

## Abstract

In para-cycling, competitors are classed based on functional impairment resulting in cyclists with neurological and locomotor impairments competing against each other. In Paralympic competition, classes are combined by using a factoring adjustment to race times to produce the overall medallists. Pacing in short-duration track cycling events is proposed to utilize an “all-out” strategy in able-bodied competition. However, pacing in para-cycling may vary depending on the level of impairment. Analysis of the pacing strategies employed by different classification groups may offer scope for optimal performance; therefore, this study investigated the pacing strategy adopted during the 1-km time trial (TT) and 500-m TT in elite C1 to C3 para-cyclists and able-bodied cyclists. Total times and intermediate split times (125-m intervals; measured to 0.001 s) were obtained from the C1-C3 men's 1-km TT (*n* = 28) and women's 500-m TT (*n* = 9) from the 2012 Paralympic Games and the men's 1-km TT (*n* = 19) and women's 500-m TT (*n* = 12) from the 2013 UCI World Track Championships from publically available video. Split times were expressed as actual time, factored time (for the para-cyclists) and as a percentage of total time. A two-way analysis of variance was used to investigate differences in split times between the different classifications and the able-bodied cyclists in the men's 1-km TT and between the para-cyclists and able-bodied cyclists in the women's 500-m TT. The importance of position at the first split was investigated with Kendall's Tau-b correlation. The first 125-m split time was the slowest for all cyclists, representing the acceleration phase from a standing start. C2 cyclists were slowest at this 125-m split, probably due to a combination of remaining seated in this acceleration phase and a high proportion of cyclists in this group being trans-femoral amputees. Not all cyclists used aero-bars, preferring to use drop, flat or bullhorn handlebars. Split times increased in the later stages of the race, demonstrating a positive pacing strategy. In the shorter women's 500-m TT, rank at the first split was more strongly correlated with final position than in the longer men's 1-km TT. In conclusion, a positive pacing strategy was adopted by the different para-cycling classes.

## Introduction

Classification for para-cycling aims to minimize the effect of impairment on competition, so that eligible athletes are grouped based on how their impairments impact the core determinants of performance (Union Cycliste Internationale., 2014). Within a classification, therefore, there may be athletes with neurological impairments (central or peripheral) and athletes with locomotor impairments. In cycling, there are currently five cycling classifications (C1-5), a tandem classification for visually-impaired cyclists (B), two tricycle classifications (T1-2), and five handbike classifications (H1-5). Of these classification types, only the cycle and tandem classes compete on the track in the velodrome. In competitions where athletes from different classifications compete in the same event, e.g., the C1-3 1-km time trial (TT) in the Paralympic Games, a factoring adjustment (usually a percentage change) according to their competing class is applied to the final times to produce the overall medallists.

Within the cycling classes, C1 contains the most affected athletes, with progressively less degree of impairment through the classes up to C5. In the C1 class, this might typically contain athletes with severe hemiplegia (lower and upper limb involvement), diplegia or severe ataxia, multiple amputation, incomplete spinal cord injuries, or muscular impairments (more than 210 points). In the C2 class, this might typically include trans-femoral amputations without the use of prosthesis when cycling, severe hemiplegia (lower limb more involved), or decreases in muscle strength (between 160 and 209 points). In the C3 class, this might typically include athletes with double trans-tibial amputations with use of prostheses, muscular/ multiple impairments (between 110 and 159 points), moderate hemiplegia or moderate ataxia. For further details on classification profiles please see the UCI regulations (Union Cycliste Internationale, 2013).

In cycling time trials, success depends on finishing in the fastest time possible. For optimal performance, all available energy stores should be used before finishing the race, without causing fatigue and a significant deceleration at the end of the race (Atkinson et al., 2007). This process of regulating energy expenditure whilst minimizing the negative consequences of fatigue through variations in momentary power output is referred to as pacing (de Koning et al., 1999; Hulleman et al., 2007). As any energy/ velocity still present after the finish line is effectively wasted kinetic energy (van Ingen Schenau et al., 1992; de Koning et al., 1999), it is suggested that an “all-out” strategy is optimal for the 1-km TT in able-bodied athletes (de Koning et al., 1999; Hulleman et al., 2007; Corbett, 2009). However, a recent laboratory study has suggested that for distances of 500-m and above, a slightly more conservative pacing pattern may be employed (de Jong et al., 2015), and peak power is dampened in trials lasting 30 and 45 s compared to 5 s indicating a central control of initial power output (Wittekind et al., 2011). Therefore, it appears that even in these short-duration trials there is evidence of a positive pacing strategy, where peak speed is achieved early in the event and then declines through the duration of the race (Abbiss and Laursen, 2008).

Events such as the 1-km and 500-m TTs are sensitive to inertial parameters and the time to reach peak speed. As these events are conducted from a stationary start position, the acceleration phase is inevitable and therefore the energy required for this phase is thought to be optimally distributed at the start of the event in a “fast start” strategy (Abbiss and Laursen, 2008). A fast-start is also associated with improved performance in short exercise bouts (3 min) due to faster $\dot{\text{V}}$O_2_ kinetics (Bailey et al., 2011). Factors such as trunk angle, hand position, and crank position all influence the initial acceleration in track cycling (Padulo et al., 2014). These factors may be constrained in para-cycling based on impairment, e.g., if an athlete is unable to start from a standing position. Therefore, depending on the nature and severity of the impairment may mean a different pacing strategy is used in para-cycling events and this might differ based on cycling class. For example, there may be reduced gross efficiency and therefore reduced exercise tolerance in amputee or neurologically impaired athletes (Hoffman et al., 1997; Sezer et al., 2004; Johnston et al., 2008; de Groot et al., 2012; Lepretre et al., 2012) compared to able-bodied athletes, and this might result in differences in pacing profile in the more affected classes.

The C1-3 classes have been reported to display noticeable inter-class performance differences in response to the 1-km TT (Lepretre et al., 2012). This was particularly apparent for the C1 compared to C2 and C3 classes, where split times increased between the C1 and other classes over the duration of the event; in contrast after a large difference in split time at 250 m, the split times were very similar between the C2 and C3 classes in the final 500 m of the event (Lepretre et al., 2012). This observation may be related to impairment differences between the classes, which may indicate that a combined category is inappropriate, or it may indicate the utilization of different pacing strategies. As the physical characteristics of the environment within the velodrome are very consistent (e.g., temperature, humidity, air resistance) within an event, split times within track racing may be used as a relatively accurate indicator of pacing strategy (Atkinson et al., 2003). Furthermore, analysis of the pacing strategies employed by the different classification groups may offer scope for optimal performance based on the degree of impairment. Therefore, the aim of this paper was to investigate the pacing strategy adopted during the 1-km TT and 500-m TT in elite C1 to C3 male and female para-cyclists. Data from able-bodied cyclists are also presented for comparison. It was hypothesized that differences in pacing profile would be apparent between able-bodied and para-cyclists, and between the different para-cycling classes.

## Materials and methods

The data were compiled from High Definition video footage available from youtube.com©. Ethics approval and informed consent were not necessary as all data are publicly available and no athlete interactions were required. Further information on the video footage analyzed is available on request from the author. Observations were made of the para-cyclist and able-bodied cyclist position during the initial acceleration from the stationary start and the position on the bike adopted during the race. Total times and 125 m split times (measured to 0.001 s; Tissot Timing, Switzerland) were obtained from the 2012 London Paralympics in the men's C1-C3 1-km TT and the women's C1-C3 500-m TT. As the 1-km and 500-m TTs were not Olympic events in 2012, data from the 2013 UCI World Track Championships in Minsk were used to provide an able-bodied comparison.

All competitor performances were included in the analysis. Twenty-eight performances were analyzed from the men's 1-km TT. Eight were in the C1 class, 9 were in the C2 class and 11 were in the C3 class. Nine performances were analyzed from the women's 500-m TT (C1 = 1, C2 = 7, C3 = 1). As the women's event had so few competitors, data was not split according to classification for the statistical analysis. Nineteen performances from the finals of the men's 1-km TT and 12 performances from the women's 500-m TT were included in the able-bodied analysis.

Observations on the start and the aerodynamic position adopted by the para-cyclists were fundamentally descriptive, so no conventional statistical analyses were conducted. Split time and total time data are presented as mean ± SD. Split times are also expressed as a percentage of final time to eliminate the effect that differences in total race time may have on the analysis, and to allow comparison with able-bodied data. Statistical analyses were conducted using SPSS version 21.0 (Chicago, IL), and statistical significance was accepted as *p* < 0.05.

A two-way analysis of variance (classification × split time), with split time as a repeated measure was used to investigate differences between pacing strategies between the different classifications for actual and factored split times. A two-way analysis of variance (classification × split time percentages), with split time percentage as a repeated measure was used to investigate differences between pacing strategies between the different classifications and the able-bodied cyclists in the men's 1-km TT and between the para-cyclists and able-bodied cyclists in the women's 500-m TT. The location of any differences were detected using a Bonferonni *post-hoc* analysis.

To gain an understanding of the importance of a relatively fast start to final position in para-cycling, the associations between the ranking at the first split time and final rankings were investigated using Kendall's tau-b test for rank correlation. Positive and negative correlations were perceived as not present/low (*T*_*B*_ < 0.50), moderate (0.50 < *T*_*B*_ < 0.70) or high (*T*_*B*_ > 0.70) (Konings et al., 2015).

## Results

In the men's event, four C1 cyclists, three C2 cyclists, and eight C3 cyclists were able to get out of the saddle at the start of the event. Additionally, five C2 cyclists (all trans-femoral amputees) appeared to be able to raise themselves off the saddle slightly on the initial down pedal stroke on their intact limb. Five C1, six C2, and all eleven C3 cyclists used aero-bars, with the remainder opting for drop, flat or bullhorn handlebars. There was some variation in aero position when using the bars, for example in how tucked the elbows were and whether both hands were transferred to the aero-bars. In contrast to the men, only one of the female cyclists used aero-bars. Five cyclists used drop handlebars, of which four cyclists were trans-femoral amputees. Three cyclists were able to get out the saddle at the start of the event. For the able-bodied cyclists, all competitors were standing for approximately the first 125 m of the TT. All 19 cyclists in the men's 1-km TT and 10 of the 12 cyclists in the women's 500-m TT used aero-bars. The two cyclists who did not use aero-bars opted for drop handlebars.

**Figure 1:**
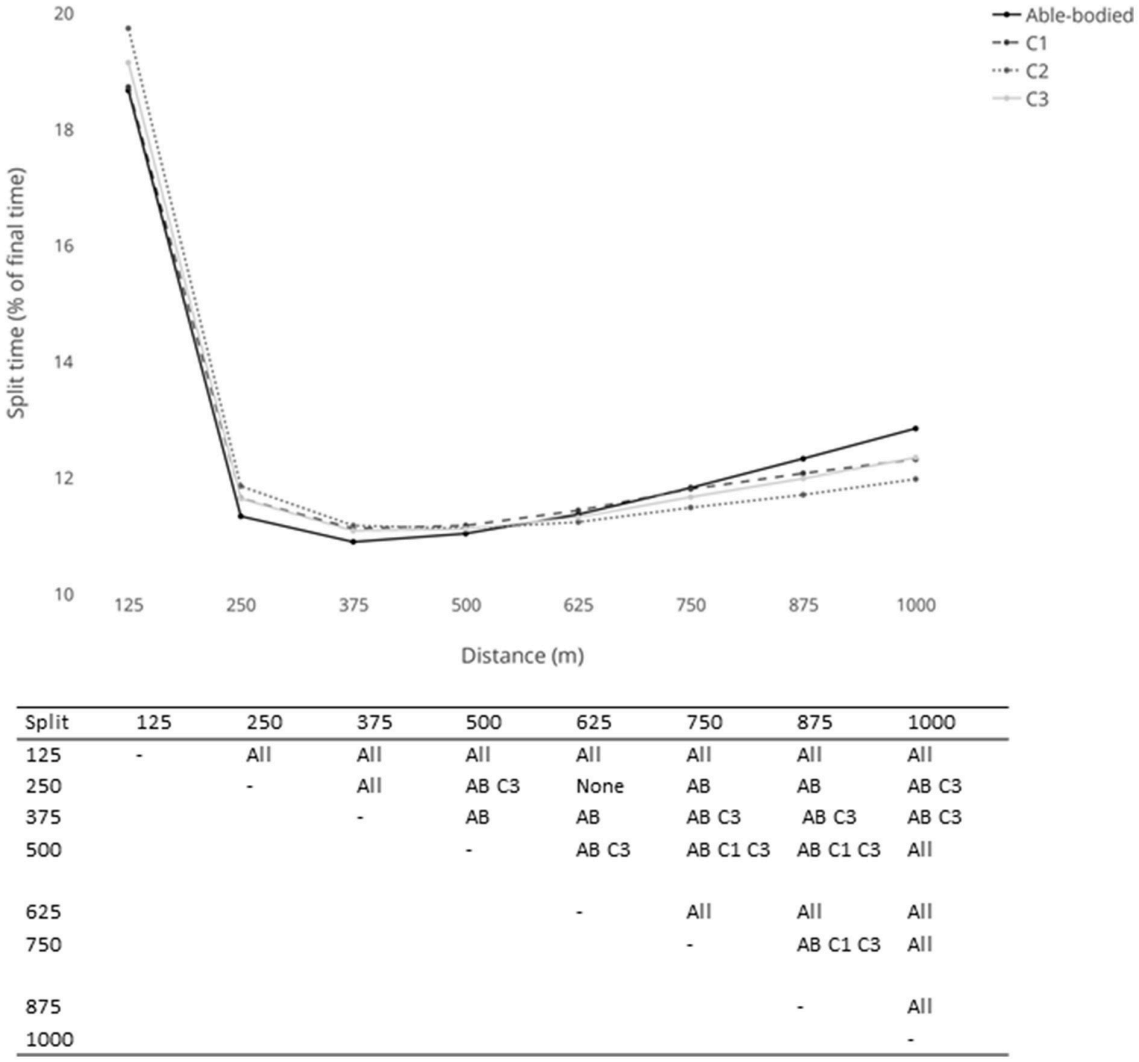
**Split times (expressed as a percentage of total time) for the men's 1-km Time Trial**. Significant differences between splits are expressed in the table below (AB, able-bodied).

The first 125 m split time was the slowest due to the acceleration from the stationary start. The third split time (250–375 m) was the fastest for the C1, C3 and able-bodied cyclists, whilst the fourth split time (375–500 m) was the fastest for the C2 cyclists. There was subsequently a progressive increase in split times for the remainder of the event (see Figure 1).

For the un-factored time splits, an interaction was found for classification type (*p* < 0.001), with differences observed between the C1 and C3 classifications (*p* < 0.001) and the C2 and C3 classifications (*p* < 0.001). When examining the interclass time differences, at the first 125 m split, the C3 cyclists started the fastest, with the C2 cyclists having the slowest split time (Table 1). By the second split (125–250 m), the C2 cyclists were recording faster split times than the C1 cyclists and recorded progressively quicker split times up until the final (875–1000 m) split. The time difference between the C1 and C3 cyclists was greatest at the first 125 m split (C3 cyclists were 1.470 s quicker), and this interclass time difference remained relatively consistent for the remainder of the event. The time difference between the C2 and C3 cyclists was also greatest at the first 125 m split (C3 cyclists were 1.717 s ahead), however this time difference was reduced further at each subsequent split with the C3 cyclists finishing 0.495 s ahead.

**Table 1:** **Split times (in seconds) as actual time and with the factored adjustment for the C1-C3 men's 1-km Time Trial and the interclass time differences (delta, s)**.

**Class**	**0–125 m**	**125–250 m**	**250–375 m**	**375–500 m**	**500–625 m**	**625–750 m**	**750–875 m**	**875–1000 m**	**Total time**
C1 (*n* = 8)	15.456 ± 1.646	9.612 ± 1.045	9.166 ± 0.842	9.209 ± 0.704	9.421 ± 0.730	9.720 ± 0.717	9.942 ± 0.720	10.141 ± 0.700	1:22.666 ± 0:06.441
C2 (*n* = 9)	15.703 ± 1.659	9.414 ± 0.569	8.863 ± 0.340	8.821 ± 0.327	8.912 ± 0.394	9.115 ± 0.447	9.283 ± 0.510	9.501 ± 0.516	1:19.612 ± 0:03.361
C3 (*n* = 11)	13.986 ± 0.741	8.494 ± 0.359	8.086 ± 0.342	8.111 ± 0.293	8.253 ± 0.293	8.511 ± 0.270	8.744 ± 0.278	9.005 ± 0.287	1:13.190 ± 0:01.994
C1-C2	−0.247	0.198	0.303	0.388	0.509	0.605	0.658	0.640	3.054
C2-C3	1.717	0.920	0.776	0.710	0.659	0.604	0.540	0.495	6.422
C1-C3	1.470	1.118	1.080	1.098	1.168	1.209	1.198	1.136	9.476
**FACTORED TIME**									
C1 (*n =* 8)	13.765 ± 1.466	8.560 ± 0.931	8.163 ± 0.750	8.202 ± 0.627	8.390 ± 0.650	8.656 ± 0.639	8.854 ± 0.641	9.031 ± 0.623	1:13.622 ± 0:05.737
C2 (*n* = 9)	14.486 ± 1.530	8.684 ± 0.524	8.176 ± 0.314	8.138 ± 0.302	8.222 ± 0.364	8.408 ± 0.413	8.563 ± 0.470	8.764 ± 0.476	1:13.442 ± 0:03.100
C3 (*n* = 11)	13.986 ± 0.741	8.493 ± 0.359	8.086 ± 0.342	8.111 ± 0.293	8.253 ± 0.293	8.511 ± 0.270	8.744 ± 0.278	9.005 ± 0.287	1:13.190 ± 0:01.994
C1-C2	−0.721	−0.124	−0.012	0.064	0.169	0.248	0.290	0.267	0.180
C2-C3	0.500	0.191	0.090	0.027	−0.032	−0.102	−0.180	−0.241	0.252
C1-C3	−0.221	0.067	0.078	0.091	0.137	0.145	0.110	0.027	0.433
**ABLE-BODIED (***n* = 19**)**									
Time	11.697 ± 0.374	7.097 ± 0.178	6.819 ± 0.177	6.905 ± 0.177	7.112 ± 0.170	7.400 ± 0.176	7.714 ± 0.207	8.042 ± 0.252	1:02.787 ± 0:01.341

*Split times (in seconds) for the able-bodied 1-km Time Trial are also presented for comparison*.

When the time splits were subjected to a factoring adjustment, there was no interaction between classification types (*p* = 0.229). When examining the interclass time differences for the factored times, the C1 cyclists recorded the fastest time at the first 125 m split, with the C2 cyclists recording the slowest (see Table 1). The factoring adjustment blunted the time differences between the classes, although as with the unadjusted times, the C2 cyclists were consistently recording faster split times than either the C1 or C3 cyclists toward the later part of the race.

There was a significant interaction between the able-bodied cyclists and the para-cyclists groups (see Figure 1). The first split was significantly slower than all other splits for all groups (*p* < 0.001). The able-bodied cyclists showed the greatest decay in split time from 375 m, whilst the C2 cyclists demonstrated a flatter pacing profile.

**Figure 2:**
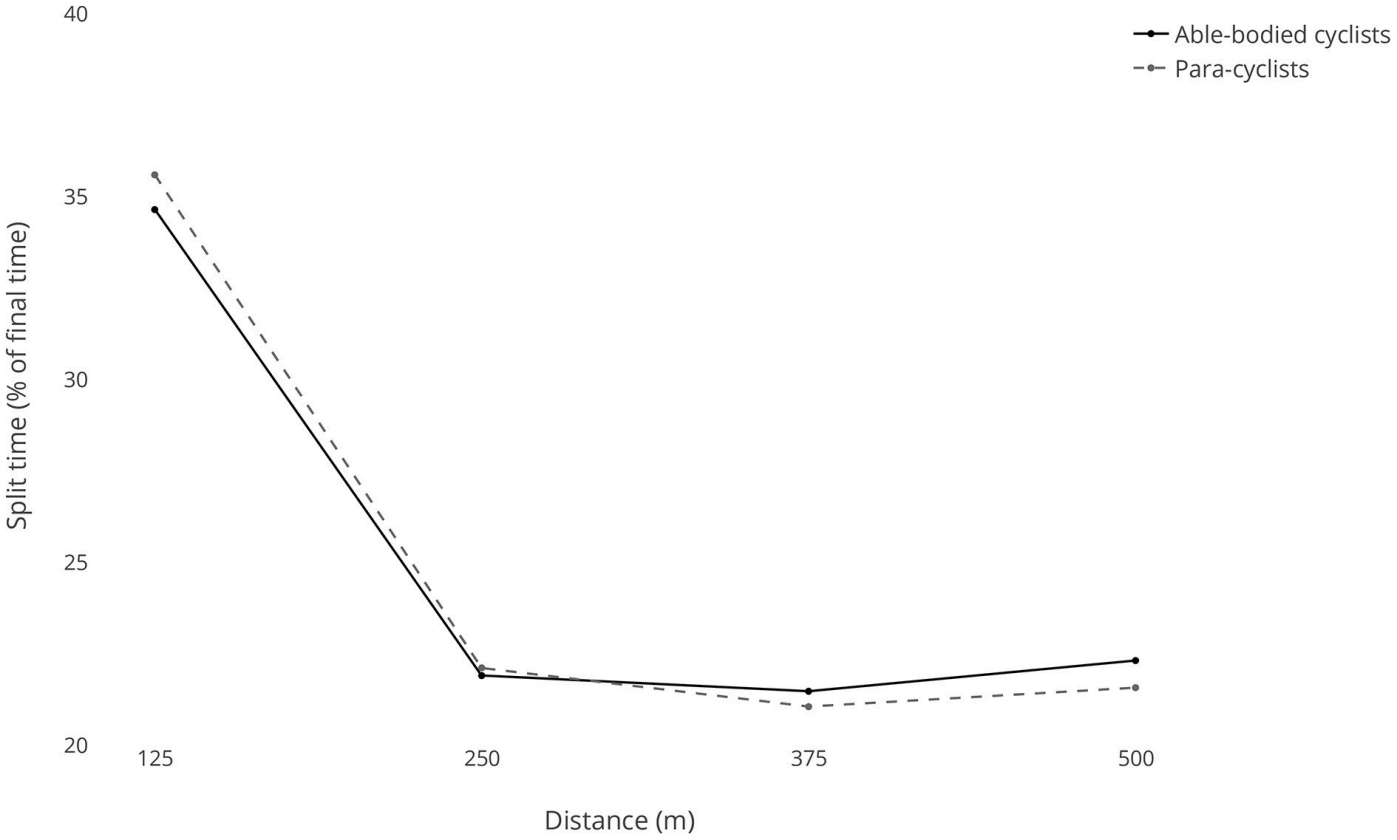
**Split times (expressed as a percentage of total time) for the women's 500-m Time Trial**.

For the women's 500-m TT, the first 125 m split was the slowest due to this being the acceleration phase from the stationary start (Table 2). The fastest split time was for the third split time (250–375 m). There was a significant interaction between the para- and able-bodied cyclists for the percentage split times (see Figure 2). For the able-bodied cyclists, all four split times were significantly different from each other (*p* < 0.001). For the para-cyclists, the first split was significantly slower (*p* < 0.001) and the third split was significantly faster (*p* < 0.05) than all other splits.

**Table 2:** **Split times (in seconds) for the para-cyclists and able-bodied cyclists in the women's 500-m Time Trial**.

	**0–125 m**	**125–250 m**	**250–375 m**	**375–500 m**	**Total time**
**PARA-CYCLISTS (***n* = 8**)**					
Split time	15.181 ± 1.404	9.410 ± 0.596	8.951 ± 0.515	9.167 ± 0.551	42.709 ± 2.724
**ABLE-BODIED (***n* = 12**)**					
Split time	11.961 ± 0.222	7.551 ± 0.146	7.401 ± 0.129	7.693 ± 0.114	34.607 ± 0.552

**Figure 3:**
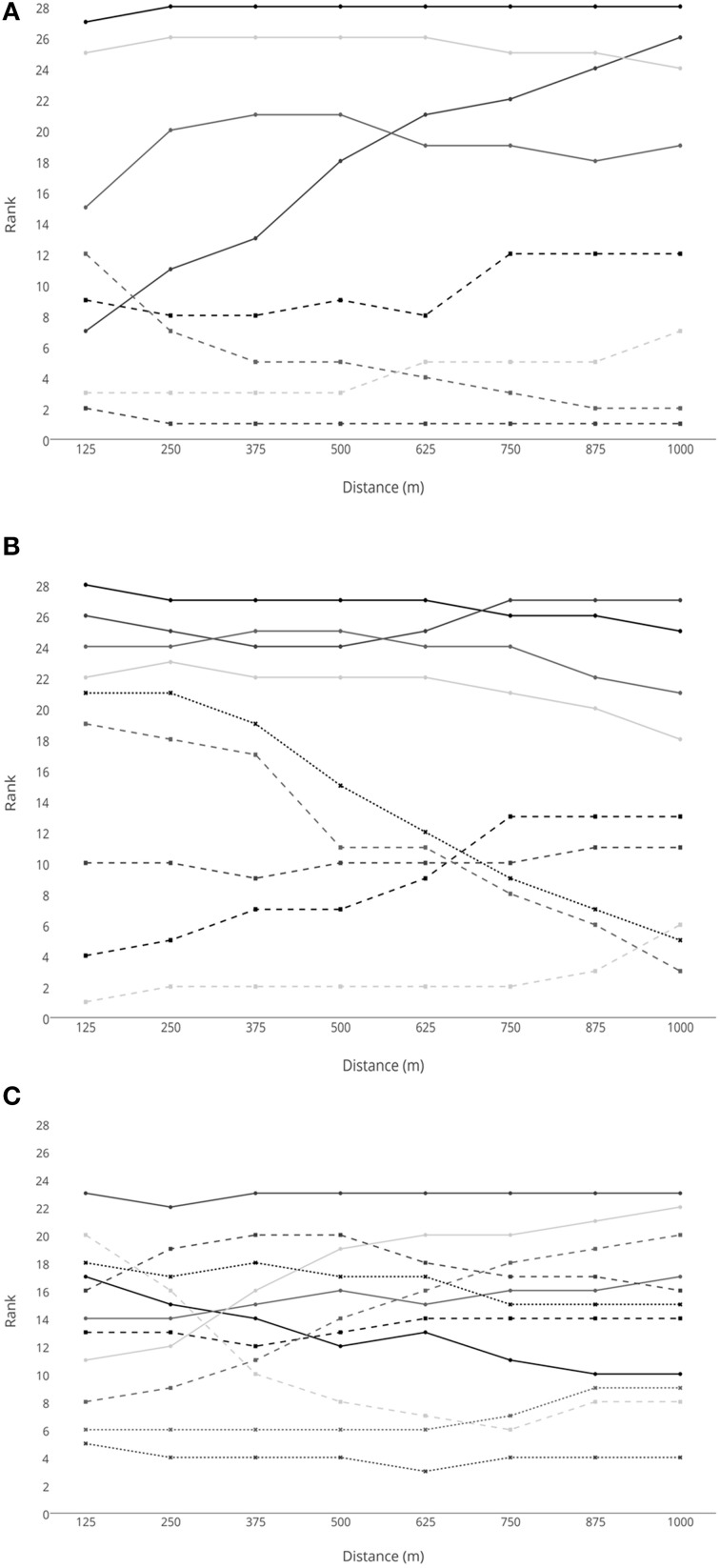
**Rank at each split in the men's 1-km Time Trial for (A) C1 cyclists, (B) C2 cyclists and (C) C3 cyclists**.

For the men's 1-km TT, there was only a low correlation between rank at the first 125 m split and final position (*T*_*B*_ = 0.413). Individual data can be observed in Figure 3. The gold medallist was ranked second at the first split but was ranked first from 250 m throughout, and also set a World Record for the C1 class. However, the silver medallist was in 12th place at the first split and the bronze medallist was in 19th position and did not enter the top 10 until 750 m into the race. The bronze medallist also set a World Record for the C2 class.

**Figure 4:**
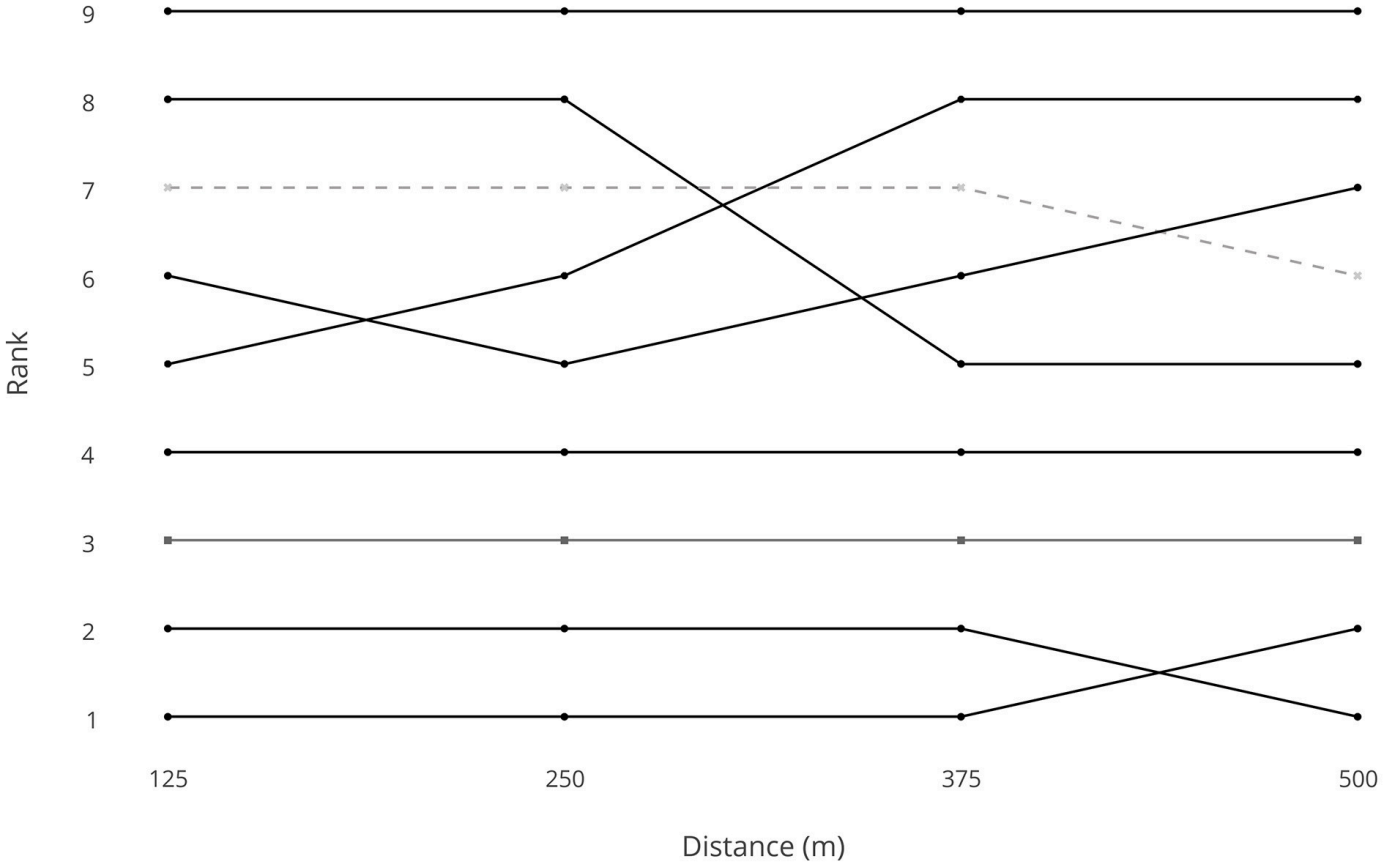
**Rank at each split in the women's 500-m Time Trial (C1 ■, C2 ●, C3 -X-)**.

For the women's 500-m TT, the cyclist's rank at the 125 m split (*T*_*B*_ = 0.611) was moderately correlated with their final position. Individual position data is visualized in Figure 4, where it can be seen that the athletes who finished in the top 4 positions were also in the top 4 positions at each split. The gold medallist set a new World Record for the C2 class, and the bronze medallist set a World Record for the C1 class.

## Discussion

The aim of this study was to investigate pacing strategies adopted by elite C1-C3 para-cyclists in the men's 1-km and women's 500-m TTs, and to compare with pacing strategies employed in elite able-bodied performance. Total time for all para-cycling classes was slower than for able-bodied cyclists at World Championship level, but similar to previously reported times for C1-C3 para-cyclists at World Championship level (Lepretre et al., 2012). In all participants, the first 125 m split time was significantly longer than all other split times. This is unsurprising as both the 1-km and 500-m TT were conducted from a stationary start, so this split time is sensitive to inertial parameters. There was a consistent significant increase in split time from 625 m onwards in the 1-km TT, and a significantly slower final split time compared to the previous split time (250–375 m) in the 500-m TT indicating an inability to maintain work output over the duration of the event. This was consistent with previous modeling (van Ingen Schenau et al., 1992; de Koning et al., 1999), laboratory (Foster et al., 2004), and competition studies (Corbett, 2009) in the 1-km TT and in laboratory studies of the 500-m TT (Foster et al., 2004) in elite able-bodied cyclists. This indicated that able-bodied cyclists and para-cyclists in these C1-3 classes adopted a positive pacing strategy.

Differences between the pacing profiles for the able-bodied and para-cyclist classes were detected in both the 1-km and 500-m TTs. The able-bodied cyclists had the lowest percentage of total time for the first 125 m split, demonstrating the able-bodied cyclists produced a relatively faster start than their para-counterparts. The able-bodied cyclists subsequently have a greater percentage of time spent in later splits in the race, demonstrating greater decay in performance. The relatively slower start for the para-cyclists is likely to be related to the impairments in these athlete populations, for example asymmetrical torque production (Brickley and Gregson, 2010) or reduced gross efficiency (Lepretre et al., 2012). Peak power is lower in cerebral palsy athletes compared to able-bodied athletes (Runciman et al., 2015), and peak power is lower in a seated compared to a standing position (Reiser et al., 2002). Peak power occurs within the first 5 s during maximal intensity cycling from a stationary start (Wright et al., 2007; Wittekind et al., 2011; O'Bryan et al., 2014), and time to peak power does not significantly change with cycling durations up to 45 s (Wittekind et al., 2011). However, as time to peak power is longer in a seated compared to standing position (Bertucci et al., 2005; Padulo et al., 2014), it is likely that for those cyclists unable to use a standing position in the initial acceleration phase at the start of the event that there is an effect on the peak power produced and the time to achieve it. Future research using power measuring cranks in para-cyclists would determine any differences in power profiles between able-bodied and para-cyclists and also between impairment types.

A significant main effect for the C1, C2, and C3 classes was apparent for the split times for the 1-km TT for the actual times. This is unsurprising as times would expect to be reduced in those who are classed as more functional during cycling. When times were factored, these differences between classes were no longer significant. This indicates that although applying a factor to times may be a blunt procedure, it does seem effective in removing the differences between classes. It is beyond the scope of this study to suggest any possible suitable alternatives for these combined-class competitions.

It has previously been suggested that larger differences were present between C1 and either C2 or C3 split times than between C2 and C3 split times in the 1-km TT, particularly as the race progressed (Lepretre et al., 2012). This progressive increase for the C1 athletes and this blunting between C2 and C3 was not present in the un-factored times in the present study. In fact, visual inspection of the split times when expressed as a percentage of total time (Table 1; Figure 1) indicated that the C1 and C3 classes show similar pacing profiles, whereas the C2 class had a greater initial 125 m split time, followed by a flatter pacing profile for the remainder of the 1-km TT. Six of the nine C2 cyclists in the 1-km TT were trans-femoral amputees. As these cyclists have to engage in single-legged cycling, they have to generate force on the pedal with just one limb throughout the whole pedal cycle. This is going to result in a lower peak power (Bundle et al., 2006), as well as an increased time to peak power through a combination of the use of one limb (Bundle et al., 2006) and being seated as opposed to standing (Bertucci et al., 2005; Padulo et al., 2014). The exercising muscle mass is required to generate more force throughout the pedal cycle in one-legged compared to two-legged cycling resulting in a higher mechanical and metabolic load (Abbiss et al., 2011). However, the differential $\dot{\text{V}}$O_2_ uptake to one- vs. two-legged exercise suggests that there may be a circulatory inhibitory response to two vs. one-legged exercise (Ogita et al., 2000), and one-legged sprint cycling relying less on anaerobic metabolism than two-legged cycling (Bundle et al., 2006), this may contribute to different fatigue profiles in the C2 class. Additionally, single-legged cycle training can result in significant improvements in the oxidative and metabolic potential of skeletal muscle in trained cyclists (Abbiss et al., 2011). Therefore, it is possible that in the 1-km TT, the slower start due to power having to be produced by a single limb can be compensated later in the event by an increased oxidative capacity for the trans-femoral amputee C2 cyclists compared to the C1 and C3 cyclists. However, previous studies on one- vs. two-legged cycling have used able-bodied cyclists; therefore the findings may not transfer to those with an amputated limb. Further research is warranted on the biomechanics and metabolic response to exercise in this population.

Aero-bars were used by five of the eight C1, six of the nine C2, all eleven C3 cyclists, and all 19 able-bodied cyclists in the men's TT, but only by one para-cyclist and 10 of the 12 able-bodied cyclists in the women's TT. Aero bars are used to reduce aerodynamic drag by reducing the frontal area. Reducing torso angle to reduce frontal area is associated with an increased crank torque but a decreased gross efficiency (Fintelman et al., 2015a). Therefore, for any cyclist the trade-off between reducing aerodynamic drag vs. decreased physiological functioning (Fintelman et al., 2015b) needs to be assessed to optimize performance particularly in a short, track TT. There was some variation in both torso angle and elbow position observed amongst the para-cyclists; whether this was determined for individual optimal performance or was due to balance and strength issues related to the individual's impairment was beyond the scope of this study. Some para-cyclists displayed balance issues when switching to the aero-bars resulting in some loss of momentum, therefore aero-bars are unlikely to be optimal for all cyclists in these categories, which may explain why some competitors have opted not to use them.

Split times from the present study indicated that although total times were slower than in elite able-bodied competition, the pacing profiles showed some similar patterns despite the mixed impairment types within the study population. In studies using elite para-cyclists, no significant differences were observed between amputee and cerebral palsy groups for $\dot{\text{V}}$O_2max_, ventilatory threshold and peak power output (Boer and Terblanche, 2014). In addition, although elite athletes with cerebral palsy displayed lower peak power outputs than able-bodied athletes during Wingate testing, the fatigue index and muscular activation was similar between the two groups (Runciman et al., 2015). Additionally, no significant differences for $\dot{\text{V}}$O_2max_ were found between physically active cerebral palsy and control participants (de Groot et al., 2012). Although these studies did not contain cyclists classed as C1, C2, or C3, the evidence indicates that elite para-athletes who have intensively trained may show a different physiology than that reported in previous studies involving untrained participants. Therefore, caution needs to be expressed extrapolating findings for specific impairments from un-trained participant studies to explain performance in elite para-sport.

In a TT there are no tactical positioning considerations that might be considered in a group race when it comes to pacing strategy (Renfree et al., 2014; Konings et al., 2015). However, by comparing position at the first split with final ranking provides insight into how relatively important it is to be the fastest early in an event in terms of final ranking, and this might provide insight into whether a fast start is optimal. In the men's 1-km TT, the rank at the first split only had a low correlation with the final position. This shows that starting faster than other competitors is not essential for determining the final medal positions at this distance. In fact, the silver medallist was in 12th place at the first split and the bronze medallist was in 19th position at the first split, and the bronze medallist did not enter the top 10 until 750 m into the race (Figure 3). Therefore, it is possible that some competitors may have accelerated too fast at the beginning of the race and therefore did not achieve optimal energy expenditure for the distance (Wilberg and Pratt, 1988). In contrast to the men's race, individual position data for the women's 500-m TT indicated that rank does not change much between the first 125 m split and the final position. In fact, the three medallists were ranked in the top 3 places throughout the TT. This indicates that starting faster than the opposition is an important factor in securing a top final position, and is consistent with shorter distances in speed skating (Muehlbauer and Schindler, 2011). As the duration of the event is short (the women's race is half the distance of the men's race), it is unlikely that the disadvantage of a slower start can be overcome before the end of the race. This may prove a disadvantage to C2 cyclists who are trans-femoral amputees if their impairment is impacting on their ability to start as fast as their competitors. Therefore, any improvement that can be gained in training, race position, and bicycle set-up to optimize power production at the start of the event is likely to benefit final position in a 500-m TT.

This study has several limitations. It was not possible to directly measure power output; therefore inferences were made regarding split times and the relationship with power output. It was assumed that peak power occurred during the rapid acceleration phase from the stationary start within the first 125 m of the event. The nature of impairment and the adaptations made to the bike may influence the aerodynamic nature of the adopted position of the cyclist which may influence cycling velocity. Finally, there were only 9 women athletes competing of whom only one was classified as C1 and one as C3. Therefore this small sample size presents a limitation to the generalization of the findings.

Future detailed research is needed on the physiology and biomechanics of para-cycling to gain further insight into the effect of different disabilities on cycling performance in elite athletes. Comparative studies between cyclists with different disabilities will inform the classification structure allowing adjustments to classes if deemed necessary. Future studies investigating responses to training in different pathologies will also inform the optimal preparation for peak performance during competition.

In conclusion, pacing strategies for para-cyclists in both the women's 500-m TT and the men's 1-km TT showed similar patterns to elite able-bodied competitors. This was characterized by a slow first split during the acceleration phase, followed by continued fast splits throughout the race. Split times increased in the later part of the race demonstrating a positive pacing strategy. Cyclists in the C2 category tended to have the slowest first split time in the men's TT, possibly due to an increased time to peak power by the trans-femoral amputee athletes in this class as they tended to remain seated during the initial acceleration phase. Starting comparatively fast and therefore being ranked highly at the first split appeared to be more closely related to final rank in the women's 500-m than the men's 1-km TT. Further research is warranted on the physiology and biomechanics of cycling in these impairment groups to inform on the demands and optimal training for improved performance.

### Conflict of interest statement

The author declares that the research was conducted in the absence of any commercial or financial relationships that could be construed as a potential conflict of interest.
